# The effect of tranexamic acid on blood loss and maternal outcome in the treatment of persistent postpartum hemorrhage: A nationwide retrospective cohort study

**DOI:** 10.1371/journal.pone.0187555

**Published:** 2017-11-06

**Authors:** Ada Gillissen, Dacia D. C. A. Henriquez, Thomas van den Akker, Camila Caram-Deelder, Merlijn Wind, Joost J. Zwart, Jos van Roosmalen, Jeroen Eikenboom, Kitty W. M. Bloemenkamp, Johanna G. van der Bom, on behalf of the TeMpOH-1 study group

**Affiliations:** 1 Centre for Clinical Transfusion Research, Sanquin Research, Leiden, the Netherlands; 2 Department of Clinical Epidemiology, Leiden University Medical Center, Leiden, The Netherlands; 3 Department of Obstetrics, Leiden University Medical Center, Leiden, The Netherlands; 4 National Perinatal Epidemiology Unit, University of Oxford, Oxford, United Kingdom; 5 Leiden University Medical Center, Leiden, The Netherlands; 6 Department of Obstetrics and Gynecology, Deventer Hospital, Deventer, The Netherlands; 7 Athena Institute, VU University Amsterdam, Amsterdam, The Netherlands; 8 Department of Thrombosis and Hemostasis, Leiden University Medical Center, Leiden, the Netherlands; 9 Department of Obstetrics, Birth Centre Wilhelmina’s Children Hospital, University Medical Center Utrecht, Utrecht, The Netherlands; Medical College of Georgia, Augusta, UNITED STATES

## Abstract

**Background:**

Recent results show a protective effect of tranexamic acid on death due to bleeding in patients with postpartum hemorrhage in low- and middle-resource countries. We quantify the association between early administration of tranexamic acid compared to late or no administration and severe acute maternal morbidity and blood loss among women suffering from persistent severe postpartum hemorrhage in a high-income country.

**Methods and findings:**

We performed a nationwide retrospective cohort study in 61 hospitals in the Netherlands. The study population consisted of 1260 women with persistent postpartum hemorrhage who had received at least four units of red cells, or fresh frozen plasma or platelets in addition to red cells. A review of medical records was performed and cross-referenced with blood bank data. The composite endpoint comprised maternal morbidity (hysterectomy, ligation of the uterine arteries, emergency B-Lynch suture, arterial embolization or admission into an intensive care unit) and mortality.

**Results:**

247 women received early tranexamic acid treatment. After adjustment for confounding, odds ratio for the composite endpoint for early tranexamic acid (n = 247) versus no/late tranexamic acid (n = 984) was 0.92 (95% confidence interval (CI) 0.66 to 1.27). Propensity matched analysis confirmed the absence of a difference between women with and without tranexamic acid. Blood loss after administration of first line therapy did not differ significantly between the two groups (adjusted difference -177 mL, CI -509.4 to +155.0).

**Conclusions:**

Our findings suggest that in a high-resource country the effect of tranexamic acid on both blood loss and the combined endpoint of maternal mortality and morbidity may be disappointing.

## Introduction

Major obstetric hemorrhage during pregnancy, delivery and puerperium continues to be an important health problem around the world. In low-resource countries, it remains the leading cause of maternal mortality. In high-resource countries it accounts for almost half of all severe acute maternal morbidity [1–4].

As part of management of major blood loss, hemostatic agents may be administered to support coagulation and correct for acquired coagulopathy [5, 6]. One of these is tranexamic acid, an antifibrinolytic agent inhibiting dissolution of the fibrin clot by binding to plasminogen and blocking the interaction of plasmin(ogen) with fibrin [7]. Tranexamic acid has been shown to reduce blood loss and the need for blood transfusion in both elective and emergency surgery [8]. Blood loss after cesarean and vaginal births was also found to be somewhat reduced by administration of tranexamic acid in absence of significant maternal and neonatal complications [9]. In severely bleeding trauma patients, tranexamic acid was found to reduce mortality by 10–15% [10]. Moreover, the CRASH-2 trial and the WOMAN trial have shown that treatment with tranexamic acid should be started as early as possible to result in the largest effect [11] [12].

In theory, postpartum hemorrhage provides an additional indication for tranexamic acid because rapid degradation of fibrinogen and fibrin and increased activation of plasminogen activators occur at placental expulsion [7, 13]. Tranexamic acid has a half-life of three hours and adequate therapeutic levels persist for 7–8 hours following intravenous injection.

Tranexamic acid is inexpensive, available in many settings, and has a good safety profile. Given the hypercoagulable status of pregnant women, possible thromboembolic side effects of tranexamic acid administration have been the subject of earlies studies. Reported adverse events were mainly minor and no clear evidence was found for an increase of thromboembolic events in pregnant women who were administered with a low dose of tranexamic acid [9]. Recently, the results of the WOMAN trial that compared tranexamic acid in an early stage of postpartum hemorrhage to placebo, showed a reduction of maternal mortality due to bleeding from 1.9% to 1.5%. However, this trial was conducted primarily in low-resource settings and no differences were found in all-cause mortality or other clinical endpoints concerning maternal morbidity. Also, the effect of tranexamic acid on the amount of blood loss was not studied. Since maternal mortality has become a rare event in high-resource countries, it remains unclear whether administration of tranexamic acid at an early stage in the course of postpartum hemorrhage has a positive effect on clinical outcome or amount of blood loss in a high-resource setting.

The aim of this study was to quantify the association between tranexamic acid administration at an early stage in the course of persistent postpartum hemorrhage and severe acute maternal morbidity and blood loss in a high-resource setting.

## Methods

### Design and study population

We performed a retrospective cohort study among women who experienced postpartum hemorrhage as part of the *Transfusion strategies in women during Major Obstetric Haemorrhage study* (TeMpOH-1). TeMpOH-1 is a nationwide retrospective cohort study in 61 hospitals in the Netherlands (75% of all hospitals in the country). The TeMpOH-1 study was approved by the Ethical Committee of the Leiden University Medical Center (P12.273) and by the institutional review board of each participating hospital. Because of the retrospective design of the study, the need to obtain informed consent was waived by the ethics committee. Data were collected retrospectively from medical files of women ≥ 18 years old, who had received four units of red blood cells or any transfusion of fresh frozen plasma (FFP) and/or platelets in addition to red blood cells because of obstetric hemorrhage between January 1^st^, 2011 and January 1^st^, 2013. Women were identified by cross-referencing electronic data from the hospitals’ blood transfusion services with local birth registers in participating hospitals. Data were recorded from medical files available at the delivery ward, operating theatre and intensive care unit for the following parameters: maternal age at the time of delivery, parity, maternal body weight during early pregnancy, maternal height, ethnicity, gestational age, obstetric history, mode of delivery, cause of major obstetric hemorrhage, abnormal placentation, shock, timing and volume of fluids and blood products transfused, medical and surgical interventions and repeated measurements of blood loss until cessation of bleeding. Blood loss was measured by weighing gauzes and all other soaked materials and by the use of a collector bag and suction system in the operating theatre. Information on possible side effects of treatment with tranexamic acid was not available due to the design of the study.

### Selection of women with persistent postpartum hemorrhage

In order to avoid use of case definitions based on mere estimations of blood loss and in absence of a universal definition of severe postpartum hemorrhage, we opted for a practically derived definition. First, we selected those women from our cohort, who had primary postpartum hemorrhage within 24 hours after birth. To meet the criteria of severe postpartum hemorrhage, bleeding had to persist despite the timely administration of first line therapy[14]. Women were treated according to the Dutch national guideline on postpartum hemorrhage, which advises to initiate first line therapy without delay in case of persisting hemorrhage despite administration of prophylactic uterotonic agents. First line therapy was defined as per primary cause of bleeding (Table 1). By applying this pragmatic definition to our cohort, only cases of ongoing hemorrhage (despite administration of first line therapy) were included for analysis.

**Table 1:** First line therapy as per primary cause of bleeding.

**Primary cause of bleeding**	**Corresponding first line therapy**
**Uterine atony**	Administration of uterotonic agents* and/ or inspection of the uterine cavity
**Retained placenta**	Manual removal of placenta
**Traumatic cause (uterine rupture, trauma of the birth canal)**	Surgical repair
**Surgical cause**	Surgical repair
**Placental abruption and placenta praevia**	Cesarean section †

* The uterotonic agents administered were Oxytocin or Misoprostol.

^†^ In case of stillbirth no cesarean section was performed

### Early versus late/no tranexamic acid

In our study, the effect of tranexamic acid was compared between patients who had received tranexamic acid early during persistent postpartum hemorrhage and patients who had not received tranexamic acid or who had received tranexamic acid at a later stage. Women who received tranexamic acid late were grouped with women who did not receive tranexamic acid at all. This allocation was chosen because at the early decision moment whether to administer tranexamic acid or not, the choice in these both groups was to refrain from administration of tranexamic acid. Thus, a control group was created of women who did not receive tranexamic acid at the early decision moment. The administration of tranexamic acid within one hour after the start of first line therapy was considered “early” administration. This cut-off point was based on the results of the CRASH-2 trial, which showed a positive effect on mortality in women who received tranexamic acid within an hour and between one and three hours after trauma. In patients who received tranexamic acid after three hours this positive effect disappeared [12,15]. Because administration of tranexamic acid in the late treatment group of the TeMpOH-2 cohort exceeded this three hours’ time limit, these women were analyzed together with the women that did not receive tranexamic acid at all.

### Outcome definitions

The primary outcome was a combined endpoint of maternal mortality and severe acute maternal morbidity. Emergency peripartum hysterectomy, ligation of the uterine arteries, B-Lynch suture (in the Netherlands only used as emergency procedure), arterial embolization or admission into an intensive care unit were considered endpoints of maternal morbidity. Secondary outcomes were total blood loss (blood loss at the end of postpartum hemorrhage), additional blood loss (additional blood loss between administration of first line therapy and the end of postpartum hemorrhage), volume and number of blood products transfused.

### Confounding and effect modifiers

The following parameters were pre-defined as possible confounders for the association between administration of tranexamic acid and outcomes: maternal age, body mass index (BMI), mode of delivery, primary cause of major obstetric hemorrhage and abnormal placentation. Also, the following bleeding characteristics at first line therapy were regarded as potential confounder: amount of blood loss, bleeding rate, shock, administration of other hemostatic agents, amount of fluids and amount of blood products that had been administered at first line therapy.

Hemorrhagic shock was defined as systolic blood pressure of 90 mmHg or less, or a heart rate of 120 bpm or higher [16]. Bleeding rate was calculated as the amount of blood loss in milliliters per minute. In the cohort, active management of labor (standard administration of oxytocin, controlled cord traction and uterine massage for prevention of postpartum hemorrhage) was practiced according to the Dutch national guidelines. (http://nvog-documenten.nl/uploaded/docs/NVOG%20richtlijn%20HPP%2014-11-2013%20herzien%202015lhe.pdf).

### Statistical analyses

Characteristics and outcomes are reported using descriptive statistics. In order to prevent bias due to missing data in covariates, we used multiple imputation. The associations between early versus no/late tranexamic acid administration and outcomes were assessed using logistic and linear regression models. The multivariate models were adjusted for measured, pre-defined confounders based on observed differences between the comparison groups.

## Results

### Patient characteristics

**Fig 1:**
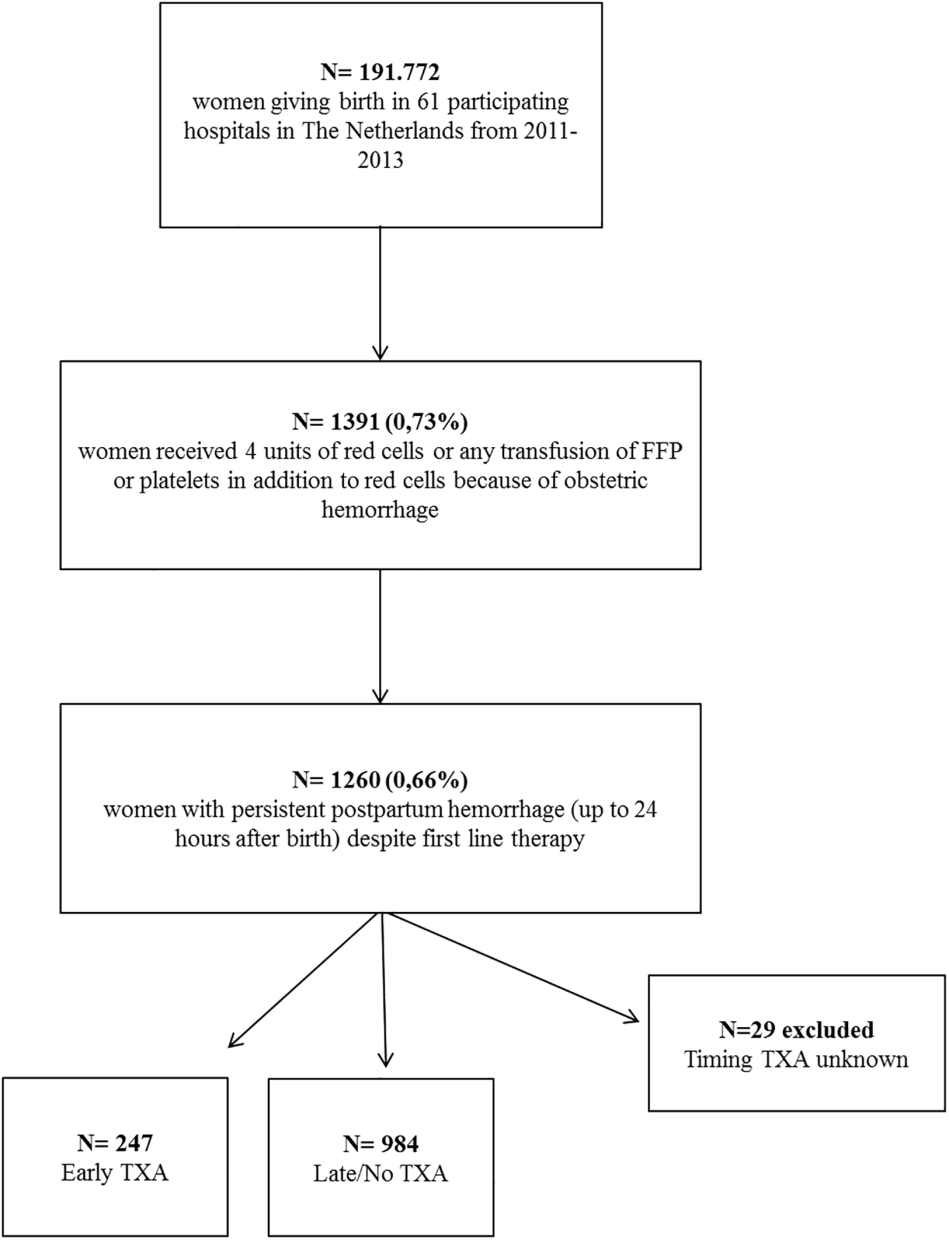
Inclusion flowchart. FFP Fresh frozen plasma, TXA Tranexamic acid.

The TeMpOH-1 cohort comprised 1391 women who had received at least four units of red cells (66%), or fresh frozen plasma or platelets in addition to red cells, for postpartum hemorrhage. For the present analysis, we selected all 1260 women who fulfilled the definition of *persistent postpartum hemorrhage*. Patient selection is presented in Fig 1. Uterine atony was the primary cause of bleeding in 789 (62%) and retained placenta in 215 (17%) women. The median total blood loss was 3000 mL (IQR 2500–4000 mL). Primary causes of bleeding and other patient characteristics are presented in Table 2. Median amount of blood loss after administration of first line therapy in the selected cohort with persistent bleeding was 2125 ml (IQR 1200–3021 ml).

**Table 2:** Patient characteristics and treatment characteristics at first line therapy according to whether women had received tranexamic acid early (within one hour after the start of first line therapy) or not early (later and no tranexamic acid).

	Early TXA n = 247	Late/No TXA n = 984
**Maternal characteristics**		
**Age**, years*	33 (29–36)	31 (28–35)
**BMI (kg/m**^**2**^)*	23 (21–26)	23 (21–27)
**Ethnicity**†		
Caucasian	181 (73)	698 (71)
Non-Caucasian	53 (21)	219 (22)
Unknown	13 (5)	67 (7)
**Obstetric Characteristics**		
**Nulliparity**† (yes)	133 (54)	511 (52)
**Gestational age** (weeks)*	40 (38–41)	40 (38–41)
**Labour characteristics**		
**Mode of delivery**†		
Vaginal	175 (71)	769 (78)
Caesarean section	71 (29)	208 (21)
**Transfer to hospital** No transfer (birth with midwife) Postpartum transfer	162 (66) 34 (14)	647 (66) 125 (13)
**Primary cause of bleeding**†		
Uterine atony	168 (68)	621 (63)
Retained placenta	39 (16)	176 (18)
Other	40 (16)	187 (19)
**Placentation**†		
Abnormal localisation placenta	34 (14)	124 (13)
Pathological ingrowth placenta	22 (9)	94 (10)
**Treatment characteristics at first line therapy**		
Fibrinogen-previously†	4 (2)	3 (0)
recombinantFVIIa-previously†	0 (0)	0 (0)
Estimated blood loss previously, ml*	1300 (450–1933)	800 (150–1400)
Bleeding rate, ml/min*	24 (14–43)	19 (9.2–38)
Shock any time before first line therapy †	76 (31)	203 (21)

* median and (IQR)

^†^ number and %

### Early versus late/no tranexamic acid administration

Of 1260 women with persistent postpartum hemorrhage 540 (42.8%) were treated with tranexamic acid, of whom 247 at an early stage, i.e. within one hour after the start of first line therapy. Among women who had received tranexamic acid early, 73% had received it at a single occasion and 21% had received tranexamic acid twice. The mean dosage per tranexamic acid gift was 1.1 grams (range 0.1 to 3.0 grams) and administration occurred intravenously. In the early treatment group, administration of tranexamic acid occurred 1.6 hours after birth (95%CI 1.3–1.9), compared to 4.6 hours (95%CI 4.1–5.1) in the late treatment group. Because of the presumed absence of benefits of administration of tranexamic acid beyond the 3-hour time interval as described in the Crash-2 trial, women in the late treatment group were analyzed together with women who did not receive tranexamic acid at all.

Table 2 presents patient characteristics according to early versus late/no tranexamic acid administration. Given the relatively large proportion of women in the Dutch maternity care system delivering with a midwife outside a hospital setting, data on location of delivery and transfer to a hospital were studied between groups. No statistically significant differences were found. Delivery by cesarean section and bleeding due to atony had occurred more frequently among women with early tranexamic acid administration, compared to women with late/no tranexamic acid administration (29% vs 21%, and 68% vs 63% respectively). In addition, women who had received tranexamic acid early, bled more severely at onset of first line therapy: bleeding rate (24 vs 19 ml/min), blood loss at diagnosis of first line therapy (1300 ml vs 800 ml) and incidence of hemorrhagic shock (31% vs 21%) were all higher compared to late/no tranexamic acid administration.

### Morbidity and mortality according to tranexamic acid administration

Six women died due to major postpartum hemorrhage (0.5%), of whom two had received early tranexamic acid. There was no clear consistent difference between women who had and those who had not received tranexamic acid early (Table 3). Adjusted odds ratio for the composite endpoint of maternal morbidity and mortality was 0.92 (95% confidence interval (CI) 0.66–1.27). There were no differences between early and late/no tranexamic acid in subsets of women with uterine atony, shock before first line therapy, blood loss above 2 liters, or by mode of delivery. (Table 4)

**Table 3:** Numbers and odds ratios (OR) of severe maternal morbidity, maternal mortality, blood loss and transfusions after first line therapy according to TXA administration.

	Early TXA n = 247	Late/No TXA n = 984	Crude OR (95% CI) or crude difference	Adjusted OR (95% CI) or difference*
Maternal mortality	2 (0,8)	4 (0,4)	2,00 (0,36–10,98)	1,31 (0,20–8,73)
Hysterectomy/ligation arteries/ B-lynch	28 (11,2)	68 (6,7)	1,71 (1,07–2,75)	1,10 (0,63–1,95)
Embolization	33 (13,1)	132 (13,1)	1,00 (0,66–1,52)	0,76 (0,48–1,22)
ICU admission	75 (29,9)	287 (28,4)	1,07 (0,78–145)	0,85 (0,60–1,19)
Composite morbidity mortality†	96 (38,2)	345 (34,2)	1,21 (0,90–1,61)	0,92 (0,66–1,27)
Additional blood loss after first line therapy (mL)‡	2150 (1058–3115)	2100 (1244–3001)	+145,7 (-167,1–458,5)	-177,2^§^ (-509,4–155,0)
Total units of RBC ǁ	4 (2–6)	4 (3–5)	0,35 (-0,20–0,90)	-0,73 (-1,35–0,10)
Total units of FFP	2 (2–4)	2 (2–3)	0,56 (0,17–0,95)	-0,12 (-0,55–0,31)
Total units of platelets	1 (1–2)	1 (1–2)	-0,03 (-0,42–0,36)	-0,40 (-0,80–0,00)
Total units of blood products	6 (4–10)	6 (4–8)	1,33 (0,33–2,32)	-0,62 (-1,72–0,48)

*ORs and differences were adjusted for pre-defined confounders: bleeding rate, measured blood loss, fluids and blood products administered at first line therapy; Occurrence of shock before first line therapy, primary cause of major obstetric haemorrhage abnormal placentation, maternal age, mode of delivery, previous administration of fibrinogen and rec FVIIa. Multiple imputation was used.

^†^ Patients that reached at least one of the predefined clinical endpoints (maternal mortality, hysterectomy/B-lynch/arterial ligation, embolization, ICU admission.

^‡^ Blood loss in ml, median & IQR are reported

^§^ Difference between groups calculated by linear regression analysis

^ǁ^ Median and IQR are reported

**Table 4:** Association between early administration of tranexamic acid versus late/no tranexamic acid and outcomes among women with atony, shock before first line therapy, volume of postpartum hemorrhage more than 2 liters, after cesarean section, or vaginal delivery.

Subgroup women with	Composite endpoint Adj. OR (95%CI)	Additional bleeding (mL) Adj. difference (95%CI)
Atony (n = 789)	0,95 (0,65–1,41)	-270 (-649–110)
Shock present before first line therapy (n = 279)	0,96 (0,52–1,79)	-105 (-662–452)
Postpartum hemorrhage > 2 liters (n = 1121)	1,71 (0,79–3,68)	-243 (-871–384)
Cesarean section (n = 279)	0,81 (0,44–1,46)	-381 (-1407–645)
Vaginal delivery (n = 944)	1,02 (0,69–1,50)	+26 (-277–329)

### Blood loss after first line therapy

The volume of additional blood loss after first line therapy was 2150 mL among women who had received tranexamic acid early whereas among women who had not received tranexamic acid early the additional blood loss was 2100 mL, amounting to a total blood loss of 3300 mL (+65% increase) vs 3000mL (+70% increase) in women who received early vs no/late tranexamic acid respectively. After adjustment for confounding the blood loss after first line therapy was slightly, but not statistically significant, lower among the women who had received early tranexamic acid (adjusted difference -177 mL, CI -509 to +155). Women who had received early tranexamic acid had been transfused 6 (IQR 4 to 10) units of blood products, which was similar to women who had not received tranexamic acid early (6 units, IQR 4 to 8).

### Sensitivity analyses

As a sensitivity analysis, we calculated propensity scores for receiving tranexamic acid early according to bleeding rate, measured blood loss, fluids and blood products administered at first line therapy, occurrence of shock before first line therapy, primary cause of major postpartum hemorrhage, abnormal placentation, maternal age, mode of delivery and previous administration of fibrinogen and recombinant FVIIa. Using nearest neighbor propensity score matching we could match 201 women who had received tranexamic acid early with women who had not received tranexamic acid early. Results of these analyses were similar to those of the main analysis. Results are provided in Table 5.

**Table 5:** Outcomes according to TXA early or late/no among women selected and matched according to the propensity score for early TXA administration.

	Early TXA n = 201	Late/No TXA n = 201	OR PS matching N = 402
Maternal mortality*	-	-	-
Hysterectomy	13 (6,5)	8 (4,0)	1,71 (0,69–4,24)
Embolization	22 (10,9)	25 (12,4)	0,87 (0,47–1,60)
ICU admission	58 (28,9)	56 (27,9)	1,06 (0,68–1,64)
Composite morbidity/Mortality	72 (35,8)	66 (32,8)	1,16 (0,76–1,77)

*Not applicable due to low numbers

## Discussion

In this nationwide study in a high-resource country, tranexamic acid administration at an early stage during the course of persistent postpartum hemorrhage was associated neither with lower maternal morbidity nor with reduced blood loss. Despite the large number of women in our cohort the confidence intervals were wide and the findings may indicate a protective as well as absence of any effect of tranexamic acid. Subgroup analyses did not reveal any particular group of women with persistent postpartum hemorrhage that might benefit from early tranexamic acid administration.

To the best of our knowledge this is the largest study to date into the effect of early treatment with tranexamic acid in patients with persistent postpartum hemorrhage in a high-resource setting. Our large sample size enables us to provide information on clinical endpoints in addition to volume of blood loss. In our study, women were included when in need of transfusion of at least 4 red blood cells or additional blood products because of persistent postpartum hemorrhage. Therefore, our results are generalizable to and may be included in meta-analyses examining women with severe postpartum hemorrhage. Some limitations to our study need to be discussed. Inherent to our observational design is bias due to confounding by indication. Such confounding bias will lead to underestimation of a possible protective effect of tranexamic acid. Despite the fact that timing of first line therapy will be similar between cases, severe cases of postpartum hemorrhage are more likely to receive early tranexamic acid as compared to less severe cases of postpartum hemorrhage. To minimize this bias, we measured and adjusted for all known and measurable confounders. In addition, we evaluated the robustness of our findings by matching women receiving early tranexamic acid with women with the same propensity scores. Nevertheless, we cannot exclude bias due to residual confounding and therefore our findings may be an underestimate of a possible truly protective effect of tranexamic acid in some women with postpartum hemorrhage. Previous studies focused on the use of tranexamic acid for *prevention* of postpartum hemorrhage, rather than treatment of postpartum hemorrhage [6]. Our results corroborate the findings of these studies. A recent systematic review and meta-analysis of RCTs that studied the effect of the *prophylactic* use of tranexamic acid for postpartum hemorrhage, showed a similar reduction in mean blood loss of 149 ml [17]. So far, few studies have looked at the effect of tranexamic acid on maternal outcome in the *treatment* of postpartum hemorrhage. The most important one is the recently published WOMAN trial, in which 20 060 women from mostly low-resource settings with blood loss above 500 ml were included. A reduction in maternal death due to bleeding from 1.9% to 1.5% was found. The largest effect occurred in the group of patients that received tranexamic acid within 1–3 hours after birth. Surprisingly no difference was found on (severe) maternal morbidity. The amount of brace sutures/ligation/embolization and blood products transfused was equal in the treatment and placebo group. And, the number of hysterectomies was higher in the tranexamic acid group. The different study settings and populations make it difficult to come to a fair comparison of the results of the studies. As also mentioned by the authors of the WOMAN trial, hysterectomy to stop postpartum hemorrhage often is a first line treatment option in low resource setting, where in a high resource setting this treatment counts as a last resort. In the WOMAN trial treatment with tranexamic acid occurred at a lower level of blood loss, which is in line with the setting of the study and the relative lack of treatment and transfusion options. Despite these differences, in both settings no significant treatment effect was found of tranexamic acid on the outcome (severe) maternal morbidity. Since maternal mortality is rare in a high resource setting, the treatment effect on mortality as found in the WOMAN trial is expected to be negligible when translated to our study population, which is in line with our results on mortality. The effect of treatment with tranexamic acid on total amount of blood loss was not studied in the WOMAN trial [18].

A French randomized controlled open-label trial studied the effect of high dose tranexamic acid (4 grams in 1 hour) versus placebo in 144 women with postpartum hemorrhage of more than 800 ml. Blood loss six hours after enrolment was lower in the tranexamic acid group, but the difference was only 48 ml, which seems clinically irrelevant [19]. A pre- and post-implementation study from the same country compared high dose tranexamic acid (4 grams in 1 hour) in 159 women with postpartum hemorrhage of more than 800 ml after vaginal birth and showed no difference in amount of blood loss, duration of bleeding or need for transfusion [20]. Both studies were rather small, and contained women with less severe postpartum hemorrhage compared to our cohort, making it difficult to assess major clinical outcomes. After the first trial some concern arose with regard to a possible association between high dosages of tranexamic acid and unexplained renal failure [20]. Treatment of postpartum hemorrhage with these high dosages of tranexamic acid was therefore discontinued in several French hospitals. Given the relatively low mean dose of tranexamic acid administered in our cohort, the chance of developing negative side effects due to tranexamic acid was considered to be low. The infusion strategy of tranexamic acid that was used in our cohort is similar to the protocol used in the Crash-2 and WOMAN trials. In the previous RCTs that evaluated the use of tranexamic acid in treatment of postpartum hemorrhage, a much higher dose (4 gram tranexamic acid in 1 hour, followed by infusion of 1 gram per hour over 6 hours) was used, based on available data on reducing hemorrhage in cardiac surgery [19]. Taking into account the pro-coagulant status of pregnancy, limiting the dosage of tranexamic acid appears to be a safer choice.

The results of our study appear plausible given the underlying mechanisms and causes of postpartum hemorrhage [21]. Administration of tranexamic acid may partially block degradation of the fibrin clot, resulting in a decrease in the amount of blood loss. However, the primary cause of bleeding in postpartum hemorrhage is in almost all cases of obstetric origin [21]. Treatment of postpartum hemorrhage should, therefore, focus on solving the underlying obstetric cause first.

This study suggests no difference or a small reduction in blood loss after administration of first line therapy in women who are treated with tranexamic acid early during persistent postpartum hemorrhage. Early treatment with tranexamic acid during persistent postpartum hemorrhage does not demonstrate a significant favorable effect on the frequency of composite maternal morbidity and mortality. Judging from the promising results of early tranexamic acid treatment in trauma medicine and elective and acute surgery and the results of the WOMAN trial, its good safety profile (when administered in dosages of 15mg/kg) and the fact that tranexamic acid is inexpensive, there seem to be few reasons not to administer tranexamic acid early during the course of persistent postpartum hemorrhage, yet the effect on clinical endpoints may be limited or absent [8, 10].

## Supporting information

S1 Dataset (SAV)
